# A randomised controlled trial of a physical activity and nutrition program targeting middle-aged adults at risk of metabolic syndrome in a disadvantaged rural community

**DOI:** 10.1186/s12889-015-1613-9

**Published:** 2015-03-25

**Authors:** Krysten Blackford, Jonine Jancey, Andy H Lee, Anthony P James, Peter Howat, Andrew P Hills, Annie Anderson

**Affiliations:** Collaboration for Evidence, Research and Impact in Public Health, Curtin University, GPO Box U1987, Perth, WA 6845 Australia; Centre for Behavioural Research in Cancer Control, Curtin University, GPO Box U1987, Perth, WA 6845 Australia; School of Public Health, Curtin University, GPO Box U1987, Perth, WA 6845 Australia; School of Exercise and Nutrition Sciences, Queensland University of Technology, GPO Box 2434, Brisbane, QLD 4001 Australia; Centre for Public Health Nutrition Research, Division of Cancer Research, Ninewells Medical School, Level 7, Mailbox 7, Dundee, DD1 9SY UK

**Keywords:** Metabolic syndrome, Nutrition, Physical activity, Middle age, Obesity, Regional/remote, Disadvantaged area, Intervention, Health promotion, Chronic disease prevention

## Abstract

**Background:**

Approximately 70% of Australian adults aged over 50 are overweight or obese, with the prevalence significantly higher in regional/remote areas compared to cities. This study aims to determine if a low-cost, accessible lifestyle program targeting insufficiently active adults aged 50-69 y can be successfully implemented in a rural location, and whether its implementation will contribute to the reduction/prevention of metabolic syndrome, or other risk factors for type 2 diabetes, and cardiovascular disease.

**Methods/Design:**

This 6-month randomised controlled trial will consist of a nutrition, physical activity, and healthy weight intervention for 50–69 year-olds from a disadvantaged rural community. Five hundred participants with central obesity and at risk of metabolic syndrome will be recruited from Albany and surrounding areas in Western Australia (within a 50 kilometre radius of the town). They will be randomly assigned to either the intervention (n = 250) or wait-listed control group (n = 250). The theoretical concepts in the study utilise the Self-Determination Theory, complemented by Motivational Interviewing. The intervention will include a custom-designed booklet and interactive website that provides information, and encourages physical activity and nutrition goal setting, and healthy weight management. The booklet and website will be supplemented by an exercise chart, calendar, newsletters, resistance bands, accelerometers, and phone and email contact from program staff. Data will be collected at baseline and post-intervention.

**Discussion:**

This study aims to contribute to the prevention of metabolic syndrome and inter- related chronic illnesses: type 2 diabetes mellitus, cardiovascular disease, and some cancers; which are associated with overweight/obesity, physical inactivity, and poor diet. This large rural community-based trial will provide guidelines for recruitment, program development, implementation, and evaluation, and has the potential to translate findings into practice by expanding the program to other regional areas in Australia.

**Trial registration:**

Australian and New Zealand Clinical Trials Registry [ACTRN12614000512628, registration date 14^th^ May 2014].

**Electronic supplementary material:**

The online version of this article (doi:10.1186/s12889-015-1613-9) contains supplementary material, which is available to authorized users.

## Background

In Australia, approximately 70% of adults aged over 50 are overweight or obese, with the prevalence significantly higher in rural areas compared to metropolitan areas [1]. The prevalence of obesity has increased 8.8% in the last 17 years with poor diet and physical inactivity likely causes [2]. Overweight/obesity coupled with advancing age increases the risk of metabolic syndrome [3], which is a cluster of abnormalities that are well documented risk factors for type 2 diabetes mellitus and cardiovascular disease [4]. These include central obesity, elevated triglyceride levels, reduced high-density lipoprotein (HDL)-cholesterol, raised blood pressure, and raised fasting plasma glucose [5].

It is estimated that approximately 25% of the Australian adult population has metabolic syndrome [3]. Prevalence is age-dependent, with the incidence rising rapidly in those of middle-age [6,7]. The relative risk of type 2 diabetes mellitus in middle-aged adults and cardiovascular disease in men has been shown to increase among those with metabolic syndrome [8]. It is imperative that overweight/obese individuals, particularly those with metabolic syndrome, are identified to ensure risk factors are addressed through lifestyle modifications and management, thereby contributing to the reduction of type 2 diabetes mellitus, cardiovascular disease, and premature death [3,6].

The prevalence of overweight/obesity has been rising among middle-aged adults living in harder to reach and often neglected rural locations of Australia [2]. These people are more likely to be overweight/obese compared with adults living in cities [9], leading to higher levels of disease risk factors. The health profile of the Great Southern region of Western Australia (location of the study area – Albany) highlights the need for greater access to health services and programs to improve the health of residents. The majority of adults (89%) do not eat the recommended daily serves of vegetables, and almost half (47.8%) do not eat adequate amounts of fruit [10], while half (50.1%) of adults do not achieve the recommended levels of physical activity [10]. In addition, Albany scores 987.4 on the Socio-Economic Indexes for Areas, indicating relative disadvantage (<1000) [1]. More disadvantaged areas have higher prevalence of risk factors and reported ill health compared with less disadvantaged areas [1].

Currently, there is a gap in the knowledge for implementing effective home-based lifestyle interventions targeting chronic disease risk reduction in disadvantaged rural areas such as Albany. This paper describes the protocol of a randomised controlled trial (RCT) that aims to address metabolic syndrome and overweight/obesity by improving the physical activity and nutrition behaviours of insufficiently active middle-aged people residing in the rural town of Albany in Western Australia. It is hypothesised that by the end of the 6-month RCT, the intervention groups compared to the control groups will show significant improvements in insulin sensitivity and lipid profile, as well as a number of anthropometric, physical activity and nutrition outcomes; thereby reducing the risk of metabolic syndrome and chronic diseases.

## Methods

### Study design

This 6-month RCT will identify and recruit insufficiently active adults aged 50 to 69 with central obesity, residing in Albany, Western Australia, to participate in a home-based physical activity, nutrition, and healthy weight management intervention. Additional file 1: Figure S1 summarises the study design. A total of 500 participants with or at risk of metabolic syndrome will be recruited and randomised into intervention (n = 250) and wait-listed control (n = 250) groups. Data will be collected from both groups at baseline and 6 months to assess changes in outcome measures. Ethical approval of the study has been obtained from the Human Research Ethics Committee of Curtin University (HR149_2013). All participants will be informed of the research aims and objectives, and that confidentiality will be maintained throughout the study. Information and consent forms will be provided prior to entry into the RCT and participants will be free to withdraw from the program at any time.

### Recruitment

Study participants aged 50–69 years and insufficiently active (less than 150 minutes of moderate physical activity per week) [11] will be recruited. Additional criteria for metabolic syndrome is based on assessing the following International Diabetes Federation (IDF) metabolic syndrome criteria [5]: central obesity (waist circumference >94 cm men or >80 cm women [Europids, Sub-Saharan Africans, Eastern Mediterranean, Middle East]; >90 cm men or >80 cm women [South Asians, Chinese, Japanese]); plus any two of: raised triglyceride level (>1.7 mM, or treatment for this); reduced HDL-cholesterol (<1.03 mM in males and <1.29 mM in females, or treatment for this); raised blood pressure (systolic ≥130 mmHg or diastolic ≥85 mmHg, or treatment of previously diagnosed hypertension); raised fasting plasma glucose (≥5.6 mM). However, only one instead of two of the latter four conditions needs to be satisfied to increase the potential pool of at risk participants. Identifying participants at risk of developing metabolic syndrome will enable appropriate intervention to be implemented, with the aim of preventing the onset of the syndrome and potential for type 2 diabetes and cardiovascular disease [12].

The following exclusion criteria will apply: previous diagnosis of diabetes mellitus (other than gestational diabetes); receiving treatment to lower blood glucose; on a weight loss diet or having weight fluctuations of >5% within the past 6 months; involvement in another physical activity and/or nutrition program; or a partner/individual residing in the same household as another participant already recruited for the study (to avoid contamination).

After the initial screening (n = 1000), it is expected that 625 participants will be eligible to join the study. Allowing for a subsequent 5% withdrawal rate and another 15% loss to follow-up, a final sample size of n = 250 intervention and n = 250 controls is anticipated, with quota sampling to ensure equal numbers of males and females. These figures are based on the pilot recruitment and data collection phase of the study.

### Procedure

Additional file 1: Figure S1 summarises the study design. Screening will occur in three stages and will be staggered to accommodate limited resources and staffing.

#### Screening stage 1

Potential participants will be initially screened by telephone via the Computer Assisted Telephone Interview system, which is an efficient method of recruitment used for a previous study [13]. During the initial contact, the purpose of the study will be explained and the caller will determine whether the individual meets the initial screening criteria. This will incorporate a number of diabetes risk factors based on AUSDRISK, an Australian type 2 diabetes mellitus risk assessment tool utilising anthropometric, lifestyle, and demographic measures [14]. This simple and reliable questionnaire will screen those at high risk of developing type 2 diabetes mellitus (risk score ≥12). Findings from our pilot data indicate that in order to achieve the required sample size, 1000 people with high risk scores will require to be screened for potential inclusion into the study.

#### Screening stage 2

After stage 1, participants meeting the initial selection criteria and indicating an interest in the study will be sent information explaining the home-based intervention project, their rights, and confidentiality details. An appointment will then be made for them to attend a central location in Albany for anthropometric measurements, blood pressure readings, and completion of a questionnaire.

#### Screening stage 3

Participants with confirmed central obesity based on screening stage 2 results will then be asked to attend a blood collection centre in Albany for the collection of a fasting blood sample (triglycerides, glucose, cholesterol) to determine whether they are at risk of metabolic syndrome.

Once eligibility criteria are determined, random assignment to intervention and control groups will occur, with quota sampling for gender. Suburbs and postcodes will be ranked according to the Socio-Economic Index for Areas and equal numbers of low and high scoring suburbs and postcodes will be allocated to each group. Intervention participants will be briefed about the purpose of the control group and the importance of refraining from communication about the program with control group members during the intervention period. They will receive the intervention materials and their AUSDRISK scores to emphasise the relevance of the program and the need for compliance to the lifestyle intervention.

### Intervention

As part of this 6-month home-based intervention, each participant will be provided with a package designed to educate, motivate, and support improvement in nutrition and levels of physical activity through goal setting, based on the principles of Self-Determination Theory and Motivational Interviewing [15,16]. The program will empower individuals to self-manage and monitor their health behaviours and weight within their own environment (home). The approach will take particular care to emphasise the importance of regular self-weighing which is widely associated with greater weight loss (showing a 1 to 3 BMI unit advantage over individuals who do not self- weight frequently) [17]. In addition, frequent self-weighing is an important behavioural mediator in weight loss maintenance [18].

The intervention program is based on recent successful physical activity and nutrition programs (Physical Activity & Nutrition for Seniors [PANS] and the Perth Active Living Seniors [PALS]) specifically designed for older adults by the research team [19,20]. It will be adapted for the younger age group and the rural context. The intervention is safe, accessible, and low cost. Participants will commence it at a low level of physical activity, with aerobic, strength and flexibility components commensurate to the fitness level of each individual. The educational materials will provide illustrations and tips on how to perform exercises safely. The physical activity component will use accelerometers to measure programmed and incidental physical activity at baseline and post-test for the intervention group only, with graphical feedback provided to participants at baseline. The nutrition component will consist of suggested meal plans, recipes, and tips on healthy eating, encouraging a higher consumption of fruit/vegetables and fibre while reducing intake of saturated fat and sugar.

Research assistants will assist with the delivery of intervention resources and liaise with the participants to provide support and to monitor their goal setting. These assistants will receive a training manual including guidelines for diet and physical activity adherence, and will undergo training in Motivational Interviewing techniques [21]. They will make telephone contact with participants at weeks 3, 6, 12, 18 and 24 of the intervention. Additionally, follow-up emails will be used, and participants will have the option to contact the research assistant they have been allocated to.

### Program resources

#### Booklet

Participants will receive a booklet designed to educate and motivate improvements in nutrition and physical activity. Content will be developed from the PANS materials [13] and adapted for a rural context. Materials will be based on the Australian Dietary Guidelines [22] and Australia’s Physical Activity and Sedentary Behaviour Guidelines [11]. Participants will be required to set their own goals for the duration of the intervention to ensure the program suits their individual needs.

#### Exercise chart, calendar, and resistance bands

The calendar will support participants’ goal setting by providing a resource for their planning, and recording their physical activity and eating habits, and will supplement the program booklet as a quick and convenient reference. Participants in the intervention group will be provided with a resistance band to use for strength training exercises. Instructions and photographs demonstrating safe and correct use will be summarised in an exercise chart [13].

#### Nutrition panel wallet cards

The wallet cards will provide assistance to participants when choosing healthy food options in a supermarket setting. The card will include information usually found on nutrition panels such as saturated fat, sugar, fibre, salt, common names for nutrients, and amounts per serve and per 100 grams considered to be healthy options.

#### Website

An online component will be developed to provide materials in electronic format to the intervention group. Pages will include nutrition and physical activity information and links, a blog for program news and updates, and a daily and weekly progress tracker enabling participants to log and track their physical activity and nutrition behaviours and monitor their weight. The website will be password protected, allowing access by the intervention group only during the study period.

#### Newsletter

A bi-monthly newsletter will be sent to intervention participants via the website, email, and post (for those without internet), to maintain participation. Previous research has shown that a 1–2 page newsletter containing health information is usually positively received in program of this nature [23,24].

### Control group

The control group (n = 250) will be placed on a ‘waitlist’ to receive the intervention after they compete the post-test data collection. This group will not receive their risk scores; however will be informed they are eligible for the study after the first group have completed their program. Previous interventions have adopted this method of wait listing the control group [25,26].

### Outcome measures

Table 1 summarises measurement of each outcome variable.

**Table 1:** **Summary of outcome variables**

***Outcome variable***	***Measuring instrument***
Metabolic syndrome diagnosis/status	IDF criteria [5]
Glucose, triglyceride, cholesterol (total, LDL, HDL, non-HDL)	Blood samples [27]
Blood pressure	OMRON automatic BP monitor
Anthropometric measurements (BMI, waist circumference, waist-to-hip ratio, body fat and muscle percentage)	Body composition scale, stadiometer, measuring tape [28,29],
Physical activity (programmed, incidental)	ActiGraph GT3X Accelerometer [30]
Sedentary and physical activity behaviours	IPAQ [13]
Diet (fat, fibre, fruit, vegetable consumption)	Fat & Fibre Barometer [31]
Economic analysis	EQ-5D-3 L [32]

*Metabolic syndrome status* will be determined using the IDF criteria [5]. Participants satisfying the full criteria will be classified as having metabolic syndrome; whereas those with central obesity (waist circumference >94 cm men or >80 cm women [Europids, Sub-Saharan Africans, Eastern Mediterranean, Middle East]; >90 cm men or >80 cm women [South Asians, Chinese, Japanese]) plus any one of: raised triglyceride level (>1.7 mM, or treatment for this); reduced HDL-cholesterol (<1.03 mM in males and <1.29 mM in females, or treatment for this); will be classified as being at risk of metabolic syndrome. Post-test reclassification will determine changes to metabolic syndrome status after 6 months.

*Blood samples* will be taken by a phlebotomist at baseline and 6 months to measure a range of markers of cardiovascular disease risk and metabolic syndrome status. Fasting plasma glucose will be measured to assess insulin sensitivity. Fasting lipid levels will also be determined to assess lipid profile and CVD risk. The concentrations of triglycerides, total cholesterol and HDL cholesterol will be measured allowing determination of total-, LDL-, HDL-, and non-HDL-cholesterol levels [27]. All blood tests will be performed by Western Diagnostic Pathology.

*Anthropometric measurements* will be undertaken by a certified anthropometrist at baseline and at 6-months post-test, following the International Society for the Advancement of Kinanthropometry guidelines. These include height and weight, body fat and muscle percentage, waist and hip circumference using a portable stadiometer, body composition scale, and tape measures, respectively [28,29]. *BMI* is promulgated as the most useful epidemiological measure of obesity along with *waist circumference*, while *waist-to-hip ratio* has been suggested as a better predictor of CVD mortality [33]. *Height* will be measured while the participant is barefoot to the nearest 0.1 cm with a portable stadiometer. *Weight* will be measured (wearing light clothing without shoes) using an electronic scale and recorded to the nearest 0.01 kg. *Waist and hip circumference* will be measured standing up at the level midway between the lowest rib margin and the iliac crest to the nearest 0.5 cm. *Hip* circumference will be measured at the largest level of the symphysis pubis and gluteus maximus. *Waist-to-hip ratio* will be calculated as waist circumference divided by hip circumference. *Blood pressure* will be measured by the trained research assistants using an Omron M5-1 electronic sphygmomanometer. A mean value will be recorded after three consecutive measurements (30).

A *self-reported questionnaire* will be completed by all participants at baseline (0-month) and post- test (6-months). This will include questions on demographics and lifestyle (smoking and alcohol drinking), the *International Physical Activity Questionnaire* (IPAQ) [13], and the *Fat & Fibre Barometer,* a valid and reliable instrument to assess dietary behavioural change [31]. The IPAQ Short Form has undergone reliability and validity testing in 12 countries. It has been used in many settings and was specifically designed for population-based physical activity studies.

*Accelerometers (ActiGraph GTX3)* will be used to objectively measure physical activity in the intervention group [30]. Participants will be asked to wear an accelerometer for 7 days at baseline and at post-test. It will be worn at the left hip throughout waking hours, removed only for showering and swimming. Data collection will be set at one-minute intervals over the day. This information will be downloaded by researchers using the ActiLife 6 software. Data will be summarised into daily average counts (counts/mins/day) and activity durations (mins/day) in specific intensity levels (inactive, light, moderate, and vigorous). A calculated weight bearing Metabolic Equivalent Task score will be determined and compared within and between groups. This will allow monitoring of incidental activity and assessment of the effect of the intervention on physical activity levels. To ensure compliance, participants will receive instructions on the device and also complete a daily log [34].

#### Economic analysis

Economic analysis of the cost-effectiveness of the intervention program will be conducted by a Health Economist. This will involve two distinct elements; firstly, a cost analysis of the physical activity and nutrition program which will provide estimates of the actual cost of the program and inferences into cost of future programs. The second aspect will include the undertaking of cost-effectiveness [35]. Quality of life will be measured using the validated EQ-5D-3 L questionnaire which is a standardised measure of health status developed by the EuroQol Group [32]. The self-complete EQ-5D-3 L questionnaire will be administered at baseline and post- test. This is a simple questionnaire which is cognitively undemanding, taking only a few minutes to complete. Sensitivity analysis will be undertaken to test the robustness of the results.

### Process evaluation

Process evaluation will assess: fidelity (quality); dose delivered (completeness); dose received (exposure, satisfaction); reach (participation rate); recruitment; and context (aspects of the environment influencing implementation or outcomes) of the intervention [36]. This will be conducted on the intervention group via a brief questionnaire administered by mail and/or internet. It will ask participants to evaluate the resources (readability, usefulness of advice, suitability and relevance to age group) based on procedures previously used by the research team [37]. Process evaluation is essential to identify the appropriateness of the intervention and research procedure.

A subgroup of the intervention participants (n = 20) will be randomly selected to take part in exit interviews. The sample size used is comparable to a previous study [13]. While the literature has identified that sample size recommendations for non-probabilistic, purposive qualitative studies can range from five to 25 participants, saturation usually occurs within the first 12 interviews [38]. Both program completers (n = 10) and non-completers (n = 10) will be contacted by telephone to gain their perceptions of the program and resources. The non-completers will be asked the reasons for withdrawal. Each interview is estimated to take between 20 and 30 minutes. Permission will be sought for recording the interviews.

### Statistical analysis

Comparisons between the changes in intervention and control groups will be performed using univariate and multivariable statistical methods. Outcome variables at baseline and post- intervention will be used to test the hypotheses in association with the covariates and confounding demographic and lifestyle variables. Continuous and categorical outcomes will be analysed using generalised linear mixed regression models, accounting for the correlations between repeated measures and clustering of the observations. Intention-to-treat analysis will be conducted to assess sensitivity of the results to the expected attrition and withdrawal of participants from the RCT.

All qualitative data will be transcribed within two weeks of interviewing. At least 10% of all data will be randomly selected and reviewed. Data will be coded and common themes or categories created. The information obtained will be collated, presented thematically and supported by direct quotes from participants. Data management of full transcripts and other text will be facilitated by NVIVO [38]. Participants’ permission will be obtained but they will not be identified in transcripts.

The economic analysis will involve both a cost analysis and cost-effectiveness analysis. This will be carried out from the perspective of the health services, including both Medicare and non- Medicare health costs, over a time horizon of 2 years. A cost analysis will provide information relating to set up, recruitment and program implementation of the RCT. While the analysis will provide specific cost details about running the RCT, the main interest will focus on the costs associated with future roll-out. Estimation will allow healthcare organisations to gauge the cost of conducting a similar program in the future.

The cost-effectiveness analysis of the trial intervention will involve the construction of a decision analytical model that captures data on both the cost and effectiveness of the intervention and non- intervention groups. The cost-effectiveness analysis will compare the difference in costs and effectiveness of the intervention and control groups, by calculating the incremental cost per Quality-Adjusted Life-Years gained by the intervention.

### Sample size

The power calculations are based on a logistic mixed regression model with the outcome variable being the prevalence of physical activity participation. Assuming 80% complete data across the assessments due to attrition and non-respondents, a total of n = 625 subjects satisfying the selection criteria will be initially recruited. In the power analyses, effect sizes of interest are associated with the time (pre-post) and intervention group parameters. For the mixed regression analysis, a final sample size of n = 500 [125 per gender by intervention condition] will provide sufficient power (80%) to detect a medium effect size at 5% significance level for the group by time interaction term accounting for gender but without other covariate adjustment.

## Discussion

### Results of the APAN study are due in late 2015

It is imperative to target middle-aged (50–69 years old) adults to address the increasing prevalence of overweight/obesity and chronic disease in Australia’s ageing population [2]. Identifying those at risk of metabolic syndrome, who are at high risk of type 2 diabetes mellitus and cardiovascular disease, can potentially lead to reduced prevalence and delayed onset of these diseases [8,39]. Providing home-based interventions for middle-aged adults still in the workforce allows for greater engagement of this group through flexibility. Flexibility provided by home-based interventions for middle-aged adults still engaged in the workforce should potentially lead to greater engagement of this hard to reach group.

Developing cost-effective strategies for identifying and managing individuals with overweight/obesity and metabolic syndrome via screening will have implications for primary care, by providing a window of opportunity for early intervention [40]. This community-based intervention will also enable large scale field testing in a rural setting, an area where little research has been conducted. Strategies evaluated as being successful and appropriate can be rolled out in other rural communities.

Cost-benefit analysis will be performed to determine the potential reduction in future chronic disease-related health costs, providing recommendations for policy and planning actions relating to the control of overweight/obesity, metabolic syndrome screening and intervention. The outcomes of the project will have potentially significant benefits to the Australian community through reduced chronic disease prevalence and improved quality of life. It will also likely have relevance to similar communities internationally.

## Additional file

Additional file 1: Figure S1. Study design.

## Abbreviations

RCT: Randomised controlled trial
BMI: Body mass index
HDL: High-density lipoprotein
APAN: Albany physical activity and nutrition
PALS: Perth active living seniors
PANS: Physical activity and nutrition for seniors
LDL: Low-density lipoprotein
