# Supplementary material for: A randomised controlled trial of a physical activity and nutrition program targeting middle-aged adults at risk of metabolic syndrome in a disadvantaged rural community
Source: BMC Public Health. 2015 Mar 25;15:284. doi: 10.1186/s12889-015-1613-9 (PMC4419409; doi:10.1186/s12889-015-1613-9)
Supplement: Additional file 1: Figure S1. — Study design. [file 12889_2015_1613_MOESM1_ESM.docx]

**Additional file 1: Figure S1: Study design**
